# Gradient of electro-convulsive therapy’s antidepressant effects along the longitudinal hippocampal axis

**DOI:** 10.1038/s41398-021-01310-0

**Published:** 2021-03-29

**Authors:** Lucien Gyger, Francesca Regen, Cristina Ramponi, Renaud Marquis, Jean-Frederic Mall, Kevin Swierkosz-Lenart, Armin von Gunten, Nicolas Toni, Ferath Kherif, Isabella Heuser, Bogdan Draganski

**Affiliations:** 1 LREN, Dept. of clinical neurosciences, Lausanne University Hospital (CHUV) and University of Lausanne, Lausanne, Switzerland; 2 Department of Psychiatry, Charité-Campus Benjamin Franklin, Berlin, Germany; 3 EEG and Epilepsy Unit, Department of Clinical Neuroscience, University Hospital of Geneva and Faculty of Medicine, Geneva, Switzerland; 4 Old Age Psychiatry service, Department of Psychiatry, Lausanne University Hospital (CHUV), and University of Lausanne, Lausanne, Switzerland; 5 Centre for Psychiatric Neurosciences, Department of Psychiatry, Lausanne University Hospital (CHUV) and Lausanne University, Lausanne, Switzerland; 6 Neurology Department, Max Planck Institute for Human Cognitive and Brain Sciences, Leipzig, Germany

**Keywords:** Neuroscience, Depression

## Abstract

Despite decades of successful treatment of therapy-resistant depression and major scientific advances in the field, our knowledge about electro-convulsive therapy’s (ECT) mechanisms of action is still scarce. Building on strong empirical evidence for ECT-induced hippocampus anatomy changes, we sought to test the hypothesis that ECT has a differential impact along the hippocampus longitudinal axis. We acquired behavioural and brain anatomy magnetic resonance imaging (MRI) data in patients with depressive episode undergoing ECT (*n* = 9) or pharmacotherapy (*n* = 24) and healthy controls (*n* = 30) at two time points 3 months apart. Using whole-brain voxel-based statistical parametric mapping and topographic analysis focused on the hippocampus, we observed ECT-induced gradient of grey matter volume increase along the hippocampal longitudinal axis with predominant impact on its anterior portion. Clinical outcome measures showed strong correlations with both baseline volume and rate of ECT-induced change exclusively for the anterior, but not posterior hippocampus. We interpret our findings confined to the anterior hippocampus and amygdala as additional evidence of the regional specific impact of ECT that unfolds its beneficial effect on depression via the “limbic” system. Main limitations of the study are patients’ polypharmacy, heterogeneity of psychiatric diagnosis, and long-time interval between scans.

## Introduction

Although the clinical efficacy of electro-convulsive therapy (ECT) for treating pharmaco-resistant depression is undisputed^1^, little is known about the underlying mechanisms of action. This renders the stratification of patients for particular ECT treatment regimens, prediction of clinical outcome, and development of strategies against ECT’s secondary effects extremely difficult. One of the established mechanistic explanations for the impact of ECT on mood is seizure-induced hippocampal neurogenesis increase^2^. The assumption of ECT-dependent neurogenesis is supported by studies in animal models^3–6^ and theoretical work, providing the link with human behaviour^7,8^. In parallel, there is a plethora of competing hypotheses about the neurobiological basis of ECT’s antidepressant effects, including changes in hippocampal glial cells number^9^, synaptogenesis^10^, or angiogenesis^11^ (reviewed in ref. ^12^).

Magnetic resonance imaging (MRI) has been extensively deployed to investigate the effects of ECT on brain and behaviour. Computational anatomy studies relying on structural MRI data and sophisticated solutions for statistical analysis in three-dimensional (3D) brain space provided consistent results of ECT-induced hippocampus volume changes (reviewed in refs. ^13,14^). With the exception of one single study^15^, all other single-centre, meta- and mega-analyses confirmed the asymmetric effect of ECT on brain structure with stronger volume increases in the right hippocampus, which shows a further increment in right unilateral stimulation compared to bilateral ECT^15–22^. The analysis of serial MRI acquisitions showed a temporal pattern of change with initial hippocampal volume gain followed either by decline to baseline or sustained volume growth. All but two studies^15,23^ have omitted to compare ECT patients with individuals treated only with pharmacotherapy, thus limiting the ability to differentiate between the effects of ECT from the impact of symptom improvement due to pharmacotherapy. Another shortcoming of computational anatomy studies using T1-weighted MRI-derived morphometrics is the inability to provide insight into ECT’s underlying neurobiological processes—neurogenesis, synaptogenesis, gliogenesis, or angiogenesis because the mechanisms driving MR signal changes in brain tissue at the microstructural level remain largely unaccounted for^24,25^.

From a neurophysiological perspective, there is a strong evidence about differentially weighted functional contribution not only between the left and right hippocampus^26,27^, but also along its transversal^28,29^ and longitudinal axes^30^. Early electro-physiological and lesion studies suggested a behavioural gradient within the hippocampus with “cold” cognitive functions—e.g., contextual memory encoding located in the posterior hippocampus and “hot” functions, e.g., emotional memory—in anterior parts^31,32^. Current research confirmed the co-existence of gradual and discrete transitions along the hippocampal longitudinal axis depending on the observational level of granularity^30^. Most recent brain imaging studies reported functional connectivity patterns from resting-state fMRI that follow a gradient of intra-subfield connectivity^33^. Up to now, there are no publications in the field of ECT that explicitly tested for a differential effect of ECT along the longitudinal hippocampal axis, although previous findings support involvement of the anterior rather than posterior hippocampus^18^ additionally to amygdala.

The main goal of our longitudinal study is to look into potentially differential effects of ECT along the longitudinal hippocampal axis. To increase the specificity of inferences, we analyse ECT-treated patients, individuals with depression receiving pharmacotherapy only and healthy controls (HC). Given the empirical evidence from previous studies and the involvement of the anterior hippocampus in limbic networks, we hypothesized that ECT will have a stronger impact in anterior compared to posterior hippocampus. To this end, we analyzed structural MRI data acquired before and after ECT treatment using a whole-brain approach in SPM12’s computational anatomy framework for longitudinal data. We further looked for ECT-induced structural changes along the hippocampal principal axes, the amygdala, and their correlation with individuals’ clinical improvement.

## Materials and methods

### Participants

We analyzed data from patients with current depressive episode and diagnosis of major depressive disorder (MDD, *n* = 22) or bipolar disorder (BD, *n* = 11) and HC (*n* = 30) included in a previous publication^23^. The sample size was chosen on the basis of previous neuroimaging studies on ECT. Our subsample differs from the reported cohort by the exclusion of bipolar patients that are in a manic episode and restricting the analysis to two time points—baseline [M0] and 3 months [M3], which allowed us to include additional study participants. Following the logic of clinical decision-making, pharmaco-resistant patients were treated with right unilateral ECT in due course of hospitalization with three ECT sessions per week (ECT; *n* = 9; 5 MDD/4 BD, 55% MDD). Patients on pharmacotherapy only (no-ECT; *n* = 24; 17 MDD/7 BD, 70% MDD) and HC (*n* = 30) followed identical data acquisition schedule. We additionally created a subgroup treated only pharmacologically that was fully matched to the MDD/BD ratio in the ECT group. Patients’ pharmacotherapy consisting of antidepressants, lithium, mood stabilizers, atypical, and typical antipsychotics was adapted during the study according to best clinical practice independently from the study participation status. For details on pharmacological therapy changes see Supplementary Table S1. Symptom severity was assessed with the 17-item Hamilton Depression Rating Scale (HAMD, score 0–50). For detailed description of the sample and between-group comparisons see Table 1. For detailed description of the subsample fully matched according to the MDD/BD ratio in the ECT group see Supplementary Table S2. This study was approved by the local ethics committee (Ethikkommission der Freien Universität Berlin im Universitätsklinikum Benjamin Franklin) and was carried out in accordance with the Declaration of Helsinki. All participants gave written informed consent before participation in the study. Healthy subjects were paid for their participation.

**Table 1:** Sociodemographic and clinical characteristics of patients treated with ECT (ECT), pharmacotherapy only (no-ECT), and healthy controls.

Group	Sociodemographic table		
	ECT	No-ECT	Healthy controls
*N*	9	24	30
Age (mean ± SD)	53.7 ± 11.1	48.9 ± 11.3	48.2 ± 11.1
Female/male	6 F/3 M	12 F/12 M	15 F/15 M
Education years (mean ± SD)	14.4 ± 2.7	14.8 ± 2.5	15.7 ± 2.2
MDD/BD	5 MDD/4 BD	17 MDD/7 BD	—
Number of depressive episode (mean ± SD)	6.9 ± 5.5	4.3 ± 3.9	—
Disease duration in years (mean ± SD)	15.4 ± 9.3	9.5 ± 10.5	—
Cumulative duration of depressive episode in months (mean ± SD)	**32.6** **±** **10.6**^a,**^	**16.3** **±** **12.5**^a,**^	—
Duration current episode in months (mean ± SD)	8.4 ± 7.2	4 ± 3.3	—
Antidepressant (%)	100%	91.7%	—
Lithium (%)	33.3%	0%	—
Mood stabilizer (%)	22.2%	25%	—
Atypical antipsychotic (%)	77.8%	29.2%	—
Typical antipsychotic (%)	0%	0%	—
HAMD at baseline (mean ± SD)	**22.9** **±** **6.1**^b,***^	**24.9** **±** **5.4**^b,***^	—
HAMD at 3 month (mean ± SD)	**8.9** **±** **7.2**^b,***^	**9.3** **±** **6.7**^b,***^	—

*MDD* major depressive disorder, *BD* bipolar disorder.

**p < 0.01, ***p < 0.001.

^a^Significant difference between ECT and no-ECT patients group.

^b^Significant difference between baseline and 3 months.

Statistically significant differences between groups are in bold.

### ECT procedure

Right unilateral ECT was administered three times a week using a square-wave, brief-pulse, constant-current device^34^. During the first ECT session, seizure threshold was individually determined by the administration of repeated stimuli of increasing intensity until a generalized seizure occurred. Stimulus intensity then was defined at 2.5 times the seizure threshold^34^. During the course of ECT stimulus intensity was further elevated in the absence of adequate seizure activity. ECT was administered with a pulse width of 0.5 ms; in two cases pulse width was temporarily shortened to 0.25 or 0.3 ms, but never exceeded 0.5 ms. After nine ECT sessions, ECT was continued on clinical judgement until response or remission was achieved. If clinically indicated, patients received continuation ECT with 12 additional ECT sessions (4 sessions weekly, 4 sessions every 2 weeks, and 4 sessions monthly). Between the two measurement time points—baseline [M0] and 3 months [M3]—patients received a mean of 21.3 ± 4.7 ECT sessions. All patients were still in the course of an ECT at 3 months, and all but one had some further ECT sessions after the second measurement time point [M3]. One patient terminated the ECT treatment 3 weeks before M3.

### MRI data acquisition and preprocessing

Structural MRI data were acquired on a 1.5 T Magnetom VISION Siemens scanner with a vacuum-moulded head holder (Vac-PacTM, Olympic Medical) for motion reduction. At each session, there were two consecutive T1-weighted acquisitions in sagittal mode using a 3D magnetization prepared rapid gradient echo (MPRAGE) sequence (TR = 11.4 ms, TE = 4.4 ms, field of view = 269 mm, flip angle = 30°, 154 contiguous slices, voxel size: 1.05 × 1.05 × 1.05 mm, slab 161 mm, matrix size = 256 × 256). We averaged the two acquisitions to obtain higher signal-to-noise-ratio. For data processing, we used SPM12 (Statistical Parametric Mapping software: www.fil.ion.ucl.ac.uk/spm, Wellcome Trust Centre for Neuroimaging, UCL London, UK) running under Matlab R2017a. Taking into account subject-specific time intervals between data acquisition time points, SPM12’s longitudinal toolbox creates midpoint average images, which represent the reference for diffeomorphic “shooting” spatial registration of all individual time point data^34,35^. Both subject-specific Jacobian determinant maps to the midpoint average were combined into a single map of diffeomorphic change. This was followed by automated classification of the midpoint average into grey matter (GM), white matter, cerebrospinal fluid and non-brain tissue probability maps in the framework of SPM12’s “unified segmentation”, using enhanced tissue priors^36^ and spatial registration to standard Montreal Neurological Institute (MNI) space. We then created maps of grey matter volume (GMV) rate of change by multiplying the subject-specific Jacobian determinant difference maps from the first step with the corresponding GM probability map from the midpoint average. The unit of the GMV map is the amount of relative change per year, i.e., for a given rate of change of 0.5 a voxel of 1 mm^3^ increases to a value of 1.5 mm^3^ after 1 year.

For the region-of-interest (ROI) analysis of hippocampus and amygdala GMV rate of change, we used the definition of hippocampal borders in the neuro-morphometric atlas in SPM12 derived from the “MICCAI 2012 Grand Challenge and Workshop on Multi-Atlas Labelling” (www.masi.vuse.vanderbilt.edu/workshop2012/index.php). As input for statistical analysis, we extracted the eigenvariate of the GMV rate of change in the anterior and posterior part of the left and right hippocampus^37,38^ (anterior *y* = −10 to −21 mm and posterior *y* = −32 to −43 mm) additionally to amygdala. We used the anterior and posterior ROI values for a three-way interaction analysis between GROUP × HEMISPHERE × SUBREGION (see Supplementary Material) and for correlation with symptom severity scores.

### Definition of hippocampal main spatial axes

For the data-driven representation of the hippocampal main spatial axes, we extracted the MNI coordinates of each hippocampal voxel within the structure defined by the neuro-morphometric atlas. We then used principal component analysis (PCA) on the *x*, *y*, and *z* coordinates and took the first principal component as indicator for the longitudinal hippocampal axis to then test for differential ECT effects along the spatial gradients (see Fig. S1).

### Statistical analysis

For statistical whole-brain analysis of ECT effects, we created a one-way analysis-of-variance design with three groups—MDD, BD, and HC, including ECT and pharmacotherapy as dummy variables additionally to regressors for age and gender. This analysis was repeated with the no-ECT group matched to the MDD/BD ratio of the ECT group.

For ROI topographical analysis with search volume restricted to the hippocampus, we used a linear mixed model with factors GROUP [ECT, no-ECT, and HC], HEMISPHERE [left and right], and AXIS [three PCA components indicative for the three main spatial axes]. To adjust for the spatial autocorrelation between voxels, we specified a 3D spherical correlation structure of the error term, using the generalized least squares approach. The correlation structure was estimated for each level of the interaction GROUP × HEMISPHERE (six correlation structures). Following the model estimation, we extracted the residuals *β* of the relationship between mean GMV rate of change and PC1 for each level of the GROUP × HEMISPHERE interaction (six *β* estimates). We then performed two series of post hoc tests where (i) each *β* was tested for significant difference from zero; (ii) estimation of the following differential contrasts: (1) *β*_ECT/Right_ vs. *β*_NoECT/Right_, (2) *β*_ECT/Right_ vs. *β*_HC/Right_, (3) *β*_NoECT/Right_ vs. *β*_HC/Right_, (4) *β*_ECT/Left_ vs. *β*_NoECT/Left_, (5) *β*_ECT/Left_ vs. *β*_HC/Left_, and (6) *β*_NoECT/Left_ vs. *β*_HC/Left_. The two families of post hoc tests were controlled for type I errors using false discovery rate (FDR) correction for multiple comparisons. This analysis was repeated with the no-ECT group matched to the MDD/BD ratio of the ECT group.

For confirmation of our parametric results, we performed a second ROI analysis using subdivision of both anterior and posterior hippocampus, as suggested previously^37^^,^^38^. We estimated a linear mixed model with between-subject fixed-effect GROUP [ECT vs. no-ECT vs. HC] and the within-subject fixed-effect HEMISPHERE [left vs. right], SUBREGION [anterior vs. posterior] after adjusting for the effects of age and sex. To account for the hierarchical nature of our data, we specified an individual-specific random intercept with all possible interactions between the factors. The planned post hoc tests with linear contrasts tested the three-way interaction GROUP × HEMISPHERE × SUBREGION—e.g., left–right difference by anterior–posterior difference by group.

We estimated the association between symptom severity (assessed with the HAMD) and baseline GMV across hippocampal SUBREGION (anterior vs. posterior) and HEMISPHERE (left vs. right), using a linear model testing the interaction with treatment group (ECT vs. no-ECT). This was repeated for the analysis of amygdala GMV. Using the same approach and design, we correlated the treatment-related symptoms severity improvement (assessed with the HAMD) and GMV rate of change in hippocampus and amygdala. Planned post hoc tests compared the difference between the slopes of the two treatment groups.

All whole-brain analyses were carried out in the general linear model framework of SPM12 using the random field theory after family-wise error (FWE) corrections for multiple comparisons at *p*_FWE_ < 0.05. For the ROIs analyses, we used the R 3.5.2 package *nlme*^39^ for fitting generalized least square and linear mixed models and the package *emmeans*^40^ for post hoc tests. We report ROI results after FDR correction for multiple comparisons.

## Results

### Demographic and clinical phenotype

There were no differences in age, sex, and years of education between groups defined by treatment—ECT (*n* = 9), no-ECT (*n* = 24), and HC (*n* = 30). The ECT and no-ECT groups did not differ in the number of depressive episodes, disease duration, and duration of the current episode, whereas the ECT group had longer cumulative duration of depressive episode compared to the no-ECT group (*p* < 0.01). Depression severity assessed with the HAMD score did not differ between ECT and no-ECT groups at any time point, i.e., both groups showed similar reduction of depression severity from baseline to 3 months (*p* < 0.001; see Table 1).

### Main effects of ECT

**Fig. 1:**
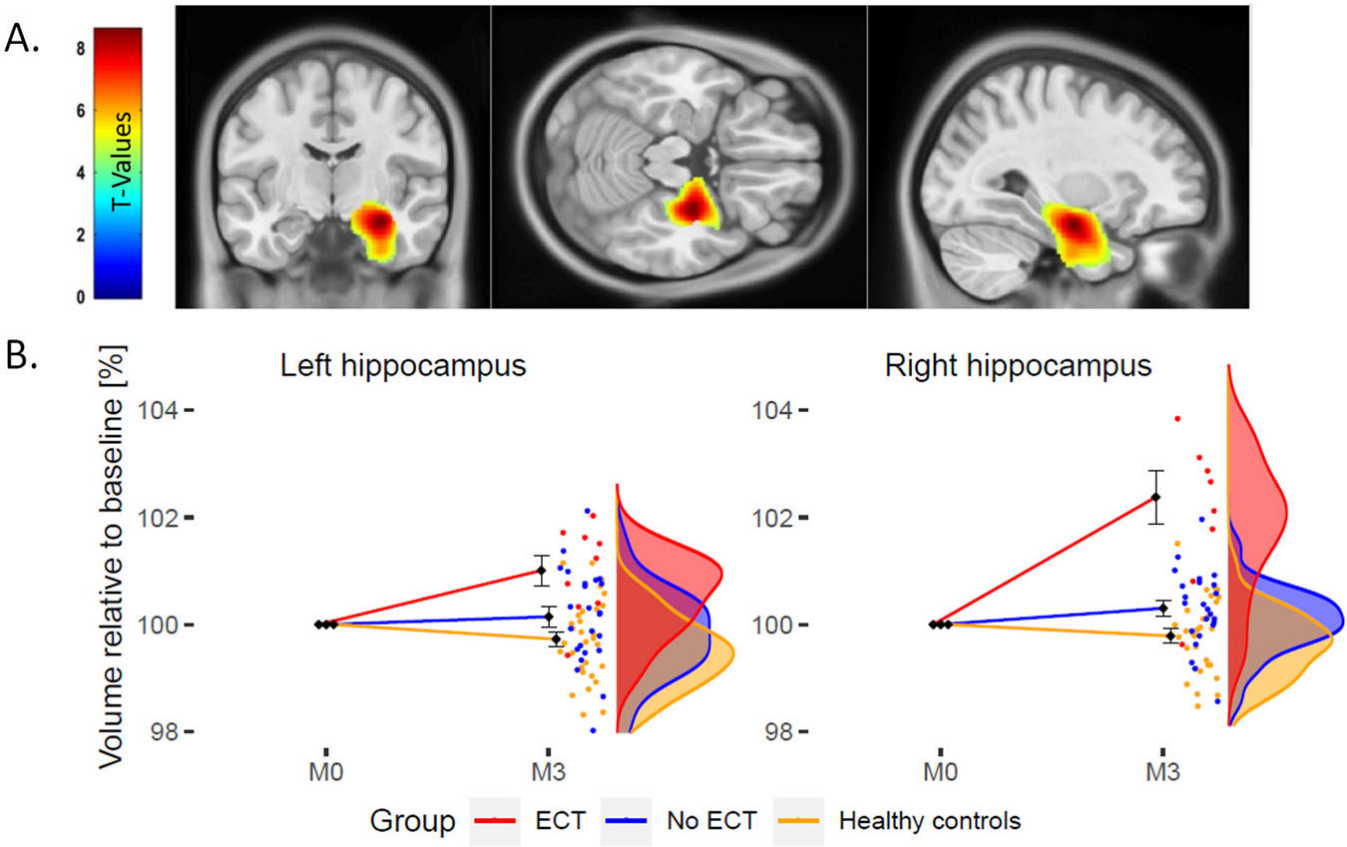
ECT-induced grey matter volume changes across the whole brain. **A**. Statistical parametric map of differential grey matter volume (GMV) rate of change in patients with electro-convulsive therapy (ECT), pharmacotherapy (no-ECT), and healthy controls (HC) projected on T1-weighted image in standard Montreal Neurological Institute space after *p*_FWE_ < 0.05 correction for multiple comparisons across the whole brain. **B** Relative volume change of left (L) and right (R) hippocampus at 3 months (M3) expressed as percentage of baseline (M0). Error bars representing standard errors.

The whole-brain voxel-based analysis showed an increase of GMV rate of change in the right hippocampal complex and amygdala for the ECT group (*p*_FWE_ < 0.05, *k* = 5183, peak: *x* = 30, *y* = −11, *z* = −20; Fig. 1A, B). We obtained similar results for the analysis, where ECT and no-ECT groups were exactly matched for the MDD/BD ratio (see Fig. S2).

**Fig. 2:**
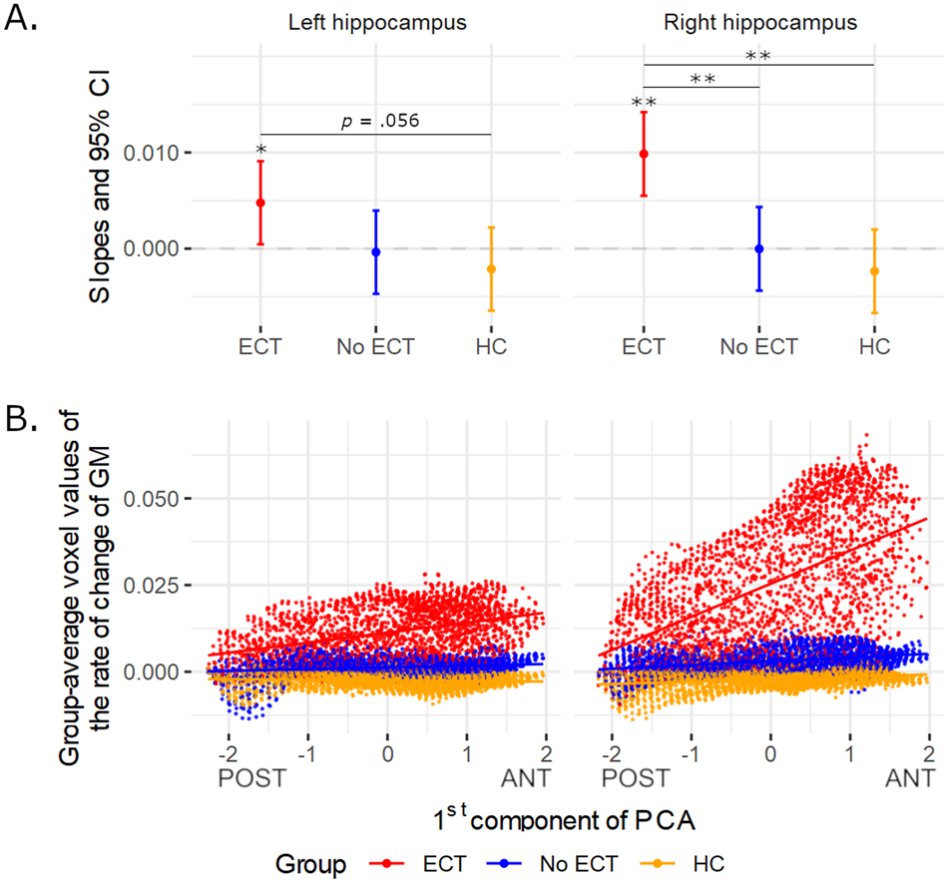
Gradient of ECT impact along the hippocampus longitudinal axis. **A** GROUP × HEMISPHERE interaction with representation of beta coefficients (with 95% CI) across GROUP (ECT—red, no-ECT—blue, and HC—yellow) after correction for multiple comparisons (*p_FDR_ < 0.05, **p_FDR_ < 0.01). **B** Correlation plot between voxel-wise grey matter (GM) volume rate of change in left and right hippocampus, and gradient along the main spatial axis of the hippocampus (first principal component) across GROUP (ECT, no-ECT, and HC). On the *x*-axis, negative value indicates voxels closer to posterior and positive value voxels closer to anterior hippocampal subregion.

The analysis for a differential ECT effect along the hippocampal antero-posterior axis demonstrated a linear increase of the GMV rate of change toward the anterior part of the hippocampus bilaterally (right: *β* = 0.01 ± 0.002, *p* < 0.01; left: *β* = 0.005 ± 0.002, *p* < 0.05, Fig. 2, and Tables S3 and 4), but not for the other interaction analyses (*p* > 0.12). The comparison of regression coefficients for the right hippocampus confirmed the steeper change in the ECT group compared with no-ECT (estimate difference = 0.01 ± 0.003, *p* < 0.01) and HC (estimate difference = 0.012 ± 0.003, *p* < 0.01). There was no difference between no-ECT and HC (estimate difference = 0.002 ± 0.003, *p* = 0.53). In the left hemisphere, we found a trend for difference between ECT and HC (estimate difference = 0.007 ± 0.003, *p* = 0.056) in the absence of other significant effects (all *p* > 0.14; Fig. 2, and Tables S3 and 4, see Fig. S3, and Tables S5 and 6 for the same analysis, but with the ratio MDD/BD matched between ECT and no-ECT groups).

The confirmatory analysis using a hard-border hippocampus subdivision showed a significant three-way GROUP × HEMISPHERE × SUBREGION interaction (*F*(2, 180) = 4, *p* < 0.05). The post hoc tests revealed that the steeper GMV rate of change in the right anterior hippocampus was specific to the ECT, when compared to the no-ECT group (estimate difference = 0.013 ± 0.005, *p* < 0.05) or the HC group (estimate difference = 0.012 ± 0.005, *p* < 0.05). There was no difference between the no-ECT and HC group (estimate difference = −0.001 ± 0.003, *p* = 0.77; Fig. S4, and Tables S7 and 8).

### Correlation with symptoms change

**Fig. 3:**
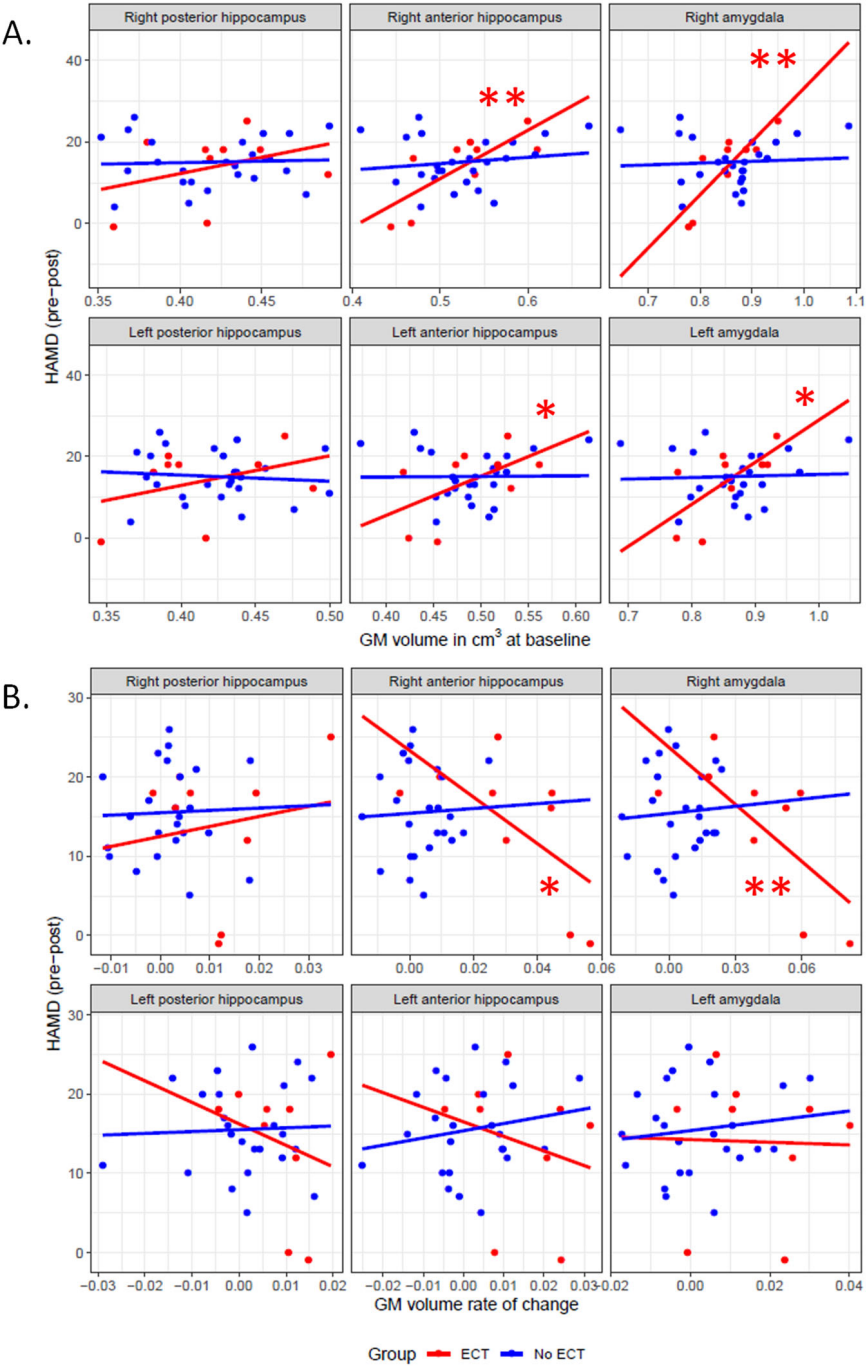
Correlation between brain anatomy and clinical scores. **A** Scatterplots of symptom improvement assessed with the Hamilton Depression Rating Scale (HAMD) vs. grey matter volume at baseline across left and right hippocampal subregions (anterior vs. posterior), and left and right amygdala after *p*_FDR_ < 0.05 correction for multiple comparisons (*) across GROUPS (ECT—red, no-ECT—blue). (*p_FDR_ < 0.05, **p_FDR_ < 0.01). **B** Scatterplots of symptom improvement assessed with the Hamilton Depression Rating Scale (HAMD) vs. grey matter volume rate of change across left and right hippocampal subregions (anterior vs. posterior), and left and right amygdala after *p*_FDR_ < 0.05 correction for multiple comparisons (*) across GROUPS (ECT and no-ECT).

We observed a positive correlation between the volume of the anterior hippocampus at baseline and symptoms change only for the ECT group (right anterior hippocampus: estimate = 118.4 ± 37.3, *p* < 0.01; left anterior hippocampus: estimate = 96.5 ± 46.9, *p* < 0.05, Fig. 3A, and Tables S9 and 10). We found the same effect in the left and right amygdala (left: estimate = 103.1 ± 38.5, *p* < 0.05; right: estimate = 130.7 ± 36.9, *p* < 0.01). The comparison of correlation slopes in the anterior hippocampus showed a steeper slope in the ECT- compared to non-ECT group for the right (estimate difference = 103 ± 43, *p* < 0.05) and a trend for the left hemisphere (estimate difference = 95 ± 55.3, *p* = 0.096). In the amygdala, the slope difference between groups was present on both sides (left: estimate difference = 103.1 ± 38.5, *p* < 0.05; right: estimate difference = 130.7 ± 36.9, *p* < 0.01).

We report a negative correlation between GMV rate of change in the right anterior hippocampus and symptoms improvement assessed with the HAMD score present only in the ECT group (estimate = −44.5 ± 17, *p* < 0.05). There was a trend toward a less steeper slope in the ECT group as compared to the no-ECT groups for the right anterior hippocampus (estimate difference = 49.06 ± 72.85, *p* = 0.09; see Fig. 3B, and Tables S11 and 12). We observed a similar pattern in the right amygdala (estimate = −36.4 ± 12.2, *p* < 0.01, estimate difference between groups = 40.9 ± 19.4, *p* < 0.05).

## Discussion

Our study provides empirical evidence for a gradient of ECT-induced behavioural and brain anatomy changes along the longitudinal hippocampal axis, with predominant effects on its anterior portion and the amygdala. We observed a steeper rate of volume change in the anterior hippocampus in ECT-treated patients compared with individuals on pharmacotherapy only and HC. There is a similar gradient linking the longitudinal hippocampal axis and amygdala anatomical characteristics with ECTs antidepressant effects, such that large anterior hippocampus and amygdala at baseline correlates with better clinical outcome after ECT, while stronger ECT-induced volume change is associated with less improvement.

The principal finding of our study is that ECT-induced brain anatomy changes follow a spatial gradient along the hippocampal longitudinal axis. To support this claim, we extended the classical inference from analysis of hippocampal ROIs to data-driven topographical estimation of ECT effects along the main spatial axes, while taking care of autocorrelation bias^41^. Our results go beyond previous observations of shape changes localized in the anterior hippocampus^18^ by adopting an iterative approach starting with whole-brain analysis that localized ECT effects to the right mesial temporal lobe followed by explicit testing for differential spatial effects along the hippocampal main axes in a restricted search volume. The convergence of findings using surface-based shape analysis^18^ and voxel-based morphometry in our study make a strong case about the regional specificity of ECT’s impact on brain anatomy. There are several lines of neurobiological interpretation that converge on the pivotal role of hippocampus in controlling stress responses via inhibition of the hypothalamic–pituitary–adrenal axis^42,43^. Depression is characterized by stress dysregulation and hippocampal atrophy^44^ and ECT thought to normalize the hyperactivity of the stress axis via seizure-induced decrease of stress hormones^45–47^. Following a reductionist view on the bipartite specialization along the hippocampus longitudinal axis, the differential structural and functional connectivity of its anterior and posterior portions support the idea of stronger involvement of the anterior hippocampus in regulating emotion and motivation^48–52^ in contrast to mnemonic processes^48,50–53^. We acknowledge that this view is a mere simplification of the complex anatomical and functional gradients along its longitudinal axis^30^.

The correlation between clinical outcome and baseline volume estimates in anterior, but not posterior hippocampus and amygdala in the ECT group lends further support to the importance of the “limbic” system for ECT’s therapeutic effects. The observation that larger anterior hippocampus and amygdala at baseline are associated with stronger symptoms reduction corroborates the findings of a recent study^54^. However, it is at odds with a previous investigation that reported the opposite pattern, although significant for the left hippocampus only^18^. The apparent controversy can stem from a number of methodological and analytical differences, the most important in our view being the reduction of a spatially dependent pattern to an average across the whole hippocampus. We denote the fine-tuned intraindividual modelling of the variable time in SPM’s longitudinal toolbox^55^ that could also explain different estimates of volume change. The correlation between clinical outcome and the magnitude of ECT-induced GMV rate of change in the anterior hippocampus lends further support to the notion of a spatial gradient of ECT effects along the hippocampal longitudinal axis. Here, the supposition of a negative relationship between the increased rate of volume change and clinical improvement contradicts the studies mentioned above^18,54^, but finds confirmation in recent meta- and mega-analyses^14,56^ that point toward an additional modulatory effect of the number of ECT treatments. This finding allows for a mechanistic interpretation under the assumption of a linear relationship between depression symptom severity, anterior hippocampus, and amygdala volume—individuals with smaller volumes at baseline show ECT-induced stronger volume increase, however less clinical benefit. This statement has to be taken with caution given the modest sample size of our cohort.

Despite the novelty of our findings, we draw attention to some limitations of our study—mainly the sample size of the ECT group and the simplification of the hippocampal anatomical axis as linear spatial construct. We also acknowledge the existence of more sophisticated methods to define the main hippocampus axis^57^. However, considering the shape of the hippocampus, we feel confident that the linear approximation of the main longitudinal axis derived from the PCA of voxel coordinates is accurate enough to capture the actual antero-posterior axis of the hippocampus. Compared to previous reports, our approach improves the signal-to-noise-ratio of the available data by averaging two MRI acquisitions per subject at each time point. The inclusion of a control group of patients is an additional strong point that helps attribute the observed effects to the ECT treatment rather than to brain anatomical changes due to symptoms improvement. Similarly, we acknowledge the limitation of our data acquisition regimen in 3 months time intervals, mainly led by a neurogenesis-focused hypothesis, which precluded assessment at the end of acute ECT treatment (typically 4–8 weeks). Given that we include pharmacologically treated patients as a natural “control” group that allows for inferring on the ECT effects as ECT-specific rather than linked to symptom improvement, we see the co-occurrence of MDD and BD, additionally to polypharmacy as potential bias against the assumption of group homogeneity.

In summary, our whole-brain analysis shows unequivocal ECT effects on the rate of volume change in the mesial temporal lobe that follow a spatial gradient along the hippocampal longitudinal axis with strongest impact on the anterior “limbic” portion and amygdala. We further highlight the importance of the notion of this spatial gradient given the correlations of the anterior hippocampus and amygdala with clinical outcome. Our findings highlight the role of the “limbic” anterior hippocampus and amygdala in unfolding the therapeutic effects of ECT, and therefore we argue that future research in this domain should consider the spatial heterogeneity not only of the hippocampus transversal axis with cytoarchitecturally well-defined borders, but also a gradient along its longitudinal axis.

## Supplementary information

Supplementary Tables and Supplementary Figures Legend

Figure S1

Figure S2

Figure S3

Figure S4
