# Supplementary material for: Gradient of electro-convulsive therapy’s antidepressant effects along the longitudinal hippocampal axis
Source: Transl Psychiatry. 2021 Mar 29;11:191. doi: 10.1038/s41398-021-01310-0 (PMC8007583; doi:10.1038/s41398-021-01310-0)
Supplement: Supplementary file 1 — Supplementary Tables and Supplementary Figures Legend [file 41398_2021_1310_MOESM1_ESM.docx]

**Supplementary Material**

**Supplementary Tables**

| **Group** | **Treatment** | **Never** | **Continuation** | **Introduction** | **Withdrawal** |
| --- | --- | --- | --- | --- | --- |
| **ECT** | ECT |  |  | 9 |  |
|  | Antidepressant |  | 8 |  | 1 |
|  | Lithium | 5 | 2 | 1 | 1 |
|  | Other Mood Stabilizer | 6 | 2 | 1 |  |
|  | Atypical antipsychotic | 1 | 5 | 1 | 2 |
|  | Typical antipsychotic | 9 |  |  |  |
| **No ECT** | ECT | 24 |  |  |  |
|  | Antidepressant | 1 | 20 | 1 | 2 |
|  | Lithium | 20 |  | 4 |  |
|  | Other Mood Stabilizer | 18 | 6 |  |  |
|  | Atypical antipsychotic | 10 | 6 | 7 | 1 |
|  | Typical antipsychotic | 23 |  | 1 |  |

**Table S1:** Summary of the change of treatment occurring during the study in the ECT and no-ECT groups.

|  | Socio-demographic table | | |
| --- | --- | --- | --- |
| N | *9* | *16* | *30* |
| Age (mean ± SD) | 53.7 ± 11.1 | 54.8 ± 8.3 | 48.2 ± 11.1 |
| Female / Male | 6F/3M | 5F/11M | 15F/15M |
| Education years (mean ± SD) | 14.4 ± 2.7 | 14.7 ± 2.5 | 15.7 ± 2.2 |
| MDD / BD | 5 MDD/4 BD | 9 MDD/7 BD | - |
| Number of depressive episode (mean ± SD) | 6.9 ± 5.5 | 5.2 ± 4.4 | - |
| Disease duration in years (mean ± SD) | 15.4 ± 9.3 | 12.3 ± 11.1 | - |
| Cumulative duration of depressive episode in months (mean ± SD) | 32.6 ± 10.6**^a(**)^** | 18.2 ± 13.7**^a(**)^** | - |
| Duration current episode in months (mean ± SD) | **8.4 ± 7.2** | **4.5 ± 3.6** | - |
| Antidepressant (%) | 1 | 0.875 | - |
| Lithium (%) | 33% | 0.0% | - |
| Mood stabilizer (%) | 22.2% | 31% | - |
| Atypical antipsychotic (%) | 77.8% | 31% | - |
| Typical antipsychotic (%) | 0.0% | 0.0% | - |
| HAMD at baseline (mean ± SD) | 22.9 ± 6.1**^b(***)^** | 24.9 ± 6.3**^b(***)^** | - |
| HAMD at 3 month (mean ± SD) | **8.9 ± 7.2^b(***)^** | **8.8 ± 7.7^b(***)^** | - |
| HAMD at 6 month (mean ± SD) | **-** | **7.3 ± 6.2** | - |
|  | ^a^significant difference between ECT and no ECTpatients group | | |
|  | bsignificant difference between baseline and 3 months | | |
|  | * = *p* < .05, ** = *p* < .01, *** = *p* < .001 | |  |

**Table S2:** Table of ECT and no-ECT cohorts matched for MDD/BD ratio.

|  | **Group** | **β estimate** | **SE** | **df** | **lower 95% CI** | **upper 95% CI** | **T-ratio** | ***p*-value** |
| --- | --- | --- | --- | --- | --- | --- | --- | --- |
| **R hippo-campus** | **ECT** | **0.0099** | **0.0021** | **7429** | **0.0055** | **0.0142** | **4.70** | **0.000** |
|  | NoECT | 0.0000 | 0.0021 | 7429 | -0.0044 | 0.0043 | -0.01 | 0.991 |
|  | HC | -0.0024 | 0.0021 | 7429 | -0.0067 | 0.0020 | -1.12 | 0.274 |
| **L hippo-campus** | **ECT** | **0.0048** | **0.0021** | **7463** | **0.0004** | **0.0091** | **2.29** | **0.032** |
|  | NoECT | -0.0004 | 0.0021 | 7463 | -0.0047 | 0.0040 | -0.18 | 0.859 |
|  | HC | -0.0021 | 0.0021 | 7463 | -0.0065 | 0.0022 | -1.02 | 0.319 |

**Table S3:** *Beta* coefficients of the generalized least square model testing the relation between grey matter volume rate of change and position along the main spatial axis of the hippocampus defined as the 1^st^ principal component of a Principal Component Analysis (PCA) performed on the MNI coordinates of the right (R) and left (L) hippocampus.

|  | **β contrast** | **SE** | **df** | **T-ratio** | ***p*-value** |
| --- | --- | --- | --- | --- | --- |
| β_ECT/Right_ - β_NoECT/Right_ | **0.0099** | **0.0030** | **7429** | **3.33** | **0.009** |
| β _ECT/Right_ - β_HC/Right_ | **0.0122** | **0.0030** | **7429** | **4.12** | **0.003** |
| β_NoECT/Right_ - β_HC/Right_ | 0.0023 | 0.0030 | 7429 | 0.79 | 0.528 |
| β_ECT/Left_ - β_NoECT/Left_ | 0.0051 | 0.0029 | 7463 | 1.75 | 0.143 |
| β_ECT/Left_ - β_HC/Left_ | 0.0069 | 0.0029 | 7463 | 2.34 | 0.058 |
| β_NoECT/Left_ - β_HC/Left_ | 0.0018 | 0.0029 | 7463 | 0.59 | 0.558 |

**Table S4:** Contrast between *beta* coefficients of the generalized least square model testing the relation between grey matter volume rate of change and the position along the main spatial axis of the hippocampus.

|  | **Group** | **β estimate** | **SE** | **df** | **lower 95% CI** | **upper 95% CI** | **T-ratio** | ***p*-value** |
| --- | --- | --- | --- | --- | --- | --- | --- | --- |
| **R hippo-campus** | **ECT** | **0.0079** | **0.0012** | **7429** | **0.0055** | **0.0103** | **6.47** | **0.000** |
|  | No ECT | 0.0002 | 0.0012 | 7429 | -0.0021 | 0.0026 | -0.24 | 0.813 |
|  | HC | -0.0011 | 0.0012 | 7429 | -0.0035 | 0.0013 | -0.91 | 0.362 |
| **L hippo-campus** | **ECT** | **0.0032** | **0.0012** | **7463** | **0.0009** | **0.0056** | **2.65** | **0.008** |
|  | No ECT | -0.0003 | 0.0012 | 7463 | -0.0027 | 0.0021 | -1.27 | 0.203 |
|  | HC | -0.0010 | 0.0012 | 7463 | -0.0034 | 0.0014 | -0.82 | 0.413 |

**Table S5:** Same table as S3 but for the analysis with reduced No-ECT group that matches the MDD/BD ratio of the ECT group.

|  | **β contrast** | **SE** | **df** | **T-ratio** | ***p*-value** |
| --- | --- | --- | --- | --- | --- |
| β_ECT/Right_ - β_NoECT/Right_ | **0.0082** | **0.0017** | **7429** | **4.74** | **0.000** |
| β _ECT/Right_ - β_HC/Right_ | **0.0090** | **0.0017** | **7429** | **5.22** | **0.000** |
| β_NoECT/Right_ - β_HC/Right_ | 0.0008 | 0.0017 | 7429 | 0.48 | 0.997 |
| β_ECT/Left_ - β_NoECT/Left_ | 0.0048 | 0.0017 | 7463 | 2.77 | 0.062 |
| β_ECT/Left_ - β_HC/Left_ | 0.0042 | 0.0017 | 7463 | 2.45 | 0.138 |
| β_NoECT/Left_ - β_HC/Left_ | -0.0006 | 0.0017 | 7463 | -0.32 | 1.000 |

**Table S6:** Same table as S4 but for the analysis with reduced No-ECT group that matches the MDD/BD ratio of the ECT group.

|  | **numDF** | **denDF** | **F-value** | **p-value** |
| --- | --- | --- | --- | --- |
| (Intercept) | 1 | 180 | 2.46 | 0.119 |
| Group | 2 | 58 | 21.37 | 0.000 |
| Subregion | 1 | 180 | 9.02 | 0.003 |
| Hemisphere | 1 | 180 | 7.90 | 0.005 |
| Age | 1 | 58 | 0.47 | 0.494 |
| Gender | 1 | 58 | 8.96 | 0.004 |
| Group x Subregion | 2 | 180 | 14.81 | 0.000 |
| Group x Side | 2 | 180 | 10.60 | 0.000 |
| Subregion x Side | 1 | 180 | 6.03 | 0.015 |
| Group x Subregion x Side | **2** | **180** | **4.00** | **0.020** |

**Table S7:** Analysis of variance - table of the confirmatory discrete analysis testing the effect of the factors GROUP x HEMISPHERE x SUBREGION with age and gender as covariates on GM volume rate of change in the hippocampus.

| **Contrast** | **Estimate** | **SE** | **df** | **T-ratio** | ***p*-value** |
| --- | --- | --- | --- | --- | --- |
| [ECT: R Ant - R Post - (L Ant - L Post)] - [NoECT: R Ant - R Post - (L Ant - L Post)] | **0.0130** | **0.0048** | **180** | **2.71** | **0.016** |
| [ECT: R Ant - R Post - (L Ant - L Post)] - [HC: R Ant - R Post - (L Ant - L Post)] | **0.0121** | **0.0047** | **180** | **2.57** | **0.016** |
| [NoECT: R Ant - R Post - (L Ant - L Post)] - [HC: R Ant - R Post - (L Ant - L Post)] | -0.0010 | 0.0034 | 180 | -0.29 | 0.768 |

**Table S8:** Post-hoc tests of the three-way interaction between GROUP x HEMISPHERE x SUBREGION of the confirmatory discrete analysis

|  | **Group** | **β estimate** | **SE** | **df** | **lower 95% CI** | **upper 95% CI** | **T-ratio** | ***p*-value** |
| --- | --- | --- | --- | --- | --- | --- | --- | --- |
| **R Post** | ECT | 80.2 | 64.3 | 30 | -51.2 | 211.5 | 1.25 | 0.222 |
|  | No ECT | 7.3 | 35.8 | 30 | -65.8 | 80.4 | 0.20 | 0.840 |
| **R Ant** | **ECT** | **118.4** | **37.3** | **30** | **42.2** | **194.6** | **3.17** | **0.003** |
|  | No ECT | 15.4 | 21.3 | 30 | -28.1 | 59.0 | 0.72 | 0.475 |
| **L Post** | ECT | 73.0 | 52.7 | 30 | -34.6 | 180.5 | 1.39 | 0.176 |
|  | No ECT | -15.1 | 38.5 | 30 | -93.6 | 63.5 | -0.39 | 0.698 |
| **L Ant** | **ECT** | **96.5** | **46.9** | **30** | **0.8** | **192.3** | **2.06** | **0.048** |
|  | No ECT | 1.6 | 29.3 | 30 | -58.2 | 61.4 | 0.05 | 0.958 |
| **R AMY** | **ECT** | **130.7** | **36.9** | **30** | **55.3** | **206.0** | **3.54** | **0.001** |
|  | No ECT | 4.4 | 14.0 | 30 | -24.2 | 33.0 | 0.31 | 0.755 |
| **L AMY** | **ECT** | **103.1** | **38.5** | **30** | **24.5** | **181.7** | **2.68** | **0.012** |
|  | No ECT | 3.7 | 18.2 | 30 | -33.5 | 40.9 | 0.21 | 0.839 |

**Table S9:** *Beta* coefficients of the regression line presented in Fig. 5 for each group and each sub-region of the right (R) and left (L) hippocampus of the four models (R Post, R Ant, L Post, L Ant) testing the relationship between the grey matter volume at baseline and symptom improvement assessed with the Hamilton depression score (HAMD) between baseline and 3 months.

| **contrast** | **β contrast** | **SE** | **df** | **T-ratio** | ***p*-value** |
| --- | --- | --- | --- | --- | --- |
| β_ECT/R Post_ - β_NoECT/R Post_ | 72.8 | 73.61 | 30 | 0.99 | 0.330 |
| β_ECT/R Ant_ - β_NoECT/R Ant_ | **103** | **42.98** | **30** | **2.40** | **0.023** |
| β_ECT/L Post_ - β_NoECT/L Post_ | 88.1 | 65.21 | 30 | 1.35 | 0.187 |
| β_ECT/L Ant_ - β_NoECT/L Ant_ | 95 | 55.27 | 30 | 1.72 | 0.096 |
| β_ECT/R AMY_ - β_NoECT/R AMY_ | **126.3** | **39.46** | **30** | **3.20** | **0.003** |
| β_ECT/L AMY_ - β_NoECT/L AMY_ | **99** | **42.58** | **30** | **2.33** | **0.026** |

**Table S10**: Group differences in each sub-region of the right (R) and left (L) hippocampus in *beta* coefficients of the four models testing the relationship between the GMV at baseline and symptom improvement assessed with the Hamilton depression score (HAMD) between baseline and 3 months (see also Fig. 5).

|  | **Group** | **β estimate** | **SE** | **df** | **lower 95% CI** | **upper 95% CI** | **T-ratio** | ***p*-value** |
| --- | --- | --- | --- | --- | --- | --- | --- | --- |
| **R Post** | ECT | 19.1 | 33.6 | 29 | -49.6 | 87.7 | 0.57 | 0.574 |
|  | No ECT | 4.5 | 28.7 | 29 | -54.2 | 63.2 | 0.16 | 0.877 |
| **R Ant** | **ECT** | **-44.5** | **17.0** | **29** | **-79.3** | **-9.6** | **-2.61** | **0.014** |
|  | No ECT | 4.6 | 22.0 | 29 | -40.4 | 49.7 | 0.21 | 0.836 |
| **L Post** | ECT | -41.0 | 49.3 | 29 | -141.9 | 59.9 | -0.83 | 0.413 |
|  | No ECT | 3.5 | 21.0 | 29 | -39.5 | 46.5 | 0.17 | 0.868 |
| **L Ant** | ECT | -27.8 | 30.0 | 29 | -89.1 | 33.5 | -0.93 | 0.361 |
|  | No ECT | 13.8 | 18.4 | 29 | -23.9 | 51.4 | 0.75 | 0.460 |
| **R AMY** | **ECT** | **-36.4** | **12.2** | **29** | **-61.4** | **-11.3** | **-2.97** | **0.006** |
|  | No ECT | 4.6 | 15.1 | 29 | -26.3 | 35.4 | 0.30 | 0.764 |
| **L AMY** | ECT | -2.6 | 25.0 | 29 | -53.6 | 48.5 | -0.10 | 0.919 |
|  | No ECT | 9.3 | 17.4 | 29 | -26.3 | 45.0 | 0.54 | 0.597 |

**Table S11:** *Beta* coefficients of the regression line presented in fig. 4 for each group and each sub-region of the right (R) and left (L) hippocampus of the four models (R Post, R Ant, L Post, L Ant) testing the relationship between the grey matter volume change and symptom improvement assessed with the Hamilton depression score (HAMD) between baseline and 3 months.

| **contrast** | **β contrast** | **SE** | **df** | **T-ratio** | ***p*-value** |
| --- | --- | --- | --- | --- | --- |
| β_ECT/R Post_ - β_NoECT/R Post_ | -14.6 | 44.16 | 29 | -0.33 | 0.744 |
| β_ECT/R Ant_ - β_NoECT/R Ant_ | 49 | 27.85 | 29 | 1.76 | 0.089 |
| β_ECT/L Post_ - β_NoECT/L Post_ | 44.5 | 53.63 | 29 | 0.83 | 0.413 |
| β_ECT/L Ant_ - β_NoECT/L Ant_ | 42 | 35.18 | 29 | 1.18 | 0.247 |
| β_ECT/R AMY_ - β_NoECT/R AMY_ | **40.9** | **19.43** | **29** | **2.11** | **0.044** |
| β_ECT/L AMY_ - β_NoECT/L AMY_ | 12 | 30.44 | 29 | 0.39 | 0.699 |

**Table S12:** Group differences in each sub-region of the L and R hippocampus and amygdala in *beta* coefficients of the four models testing the relationship between the grey matter volume change and symptom improvement assessed with the Hamilton depression score (HAMD) between baseline and 3 months (relative to Fig. 4).

**Figures**

**Figure S1:** Principal component analysis of right (R) and left (L) hippocampus coordinates corresponding to Montreal Neurological Institute (MNI) standard space. Red arrows represent the main axes estimation resulting from the Principal Component Analysis (PCA) for the right hippocampus.

**Figure S2:** **A.** Statistical Parametric Map of differential grey matter volume (GMV) rate of change in patients with electro-convulsive therapy (ECT), pharmacotherapy (no-ECT group with ratio MDD/BD matched with ECT group) and healthy controls (HC) projected on T1-weighted image in standard Montreal Neurological Institute space after p_FWE_ < .05 correction for multiple comparisons across the whole-brain. **B.** Contrast estimate and 90% Confidence Interval for each of the sub-groups separated by diagnoses.

**Figure S3: A.** GROUP x HEMISPHERE interaction with representation of beta coefficients (with 95% CI) across GROUP (ECT - red, no-ECT - blue and HC - yellow) after correction for multiple comparisons (* p_FDR_ < .05, ** p_FDR_ < .01). **B.** Correlation plot between voxel-wise grey matter (GM) volume rate of change in left and right hippocampus and gradient along the main spatial axis of the hippocampus (1^st^ principal component) across GROUP (ECT, no-ECT and HC). On the x-axis, negative value indicates voxels closer to posterior and positive value voxels closer to anterior hippocampal sub-region.

**Figure S4:** Three-way GROUP x HEMISPHERE x SUBREGION interaction. Each contrast is testing the between group difference of the right (R) vs. left (L) within group difference in subregional rate of change of grey matter volume after p_FDR_ <.05 correction for multiple comparisons (*).
