# Supplementary figures and images for: Gradient of electro-convulsive therapy’s antidepressant effects along the longitudinal hippocampal axis
Source: Transl Psychiatry. 2021 Mar 29;11:191. doi: 10.1038/s41398-021-01310-0 (PMC8007583; doi:10.1038/s41398-021-01310-0)

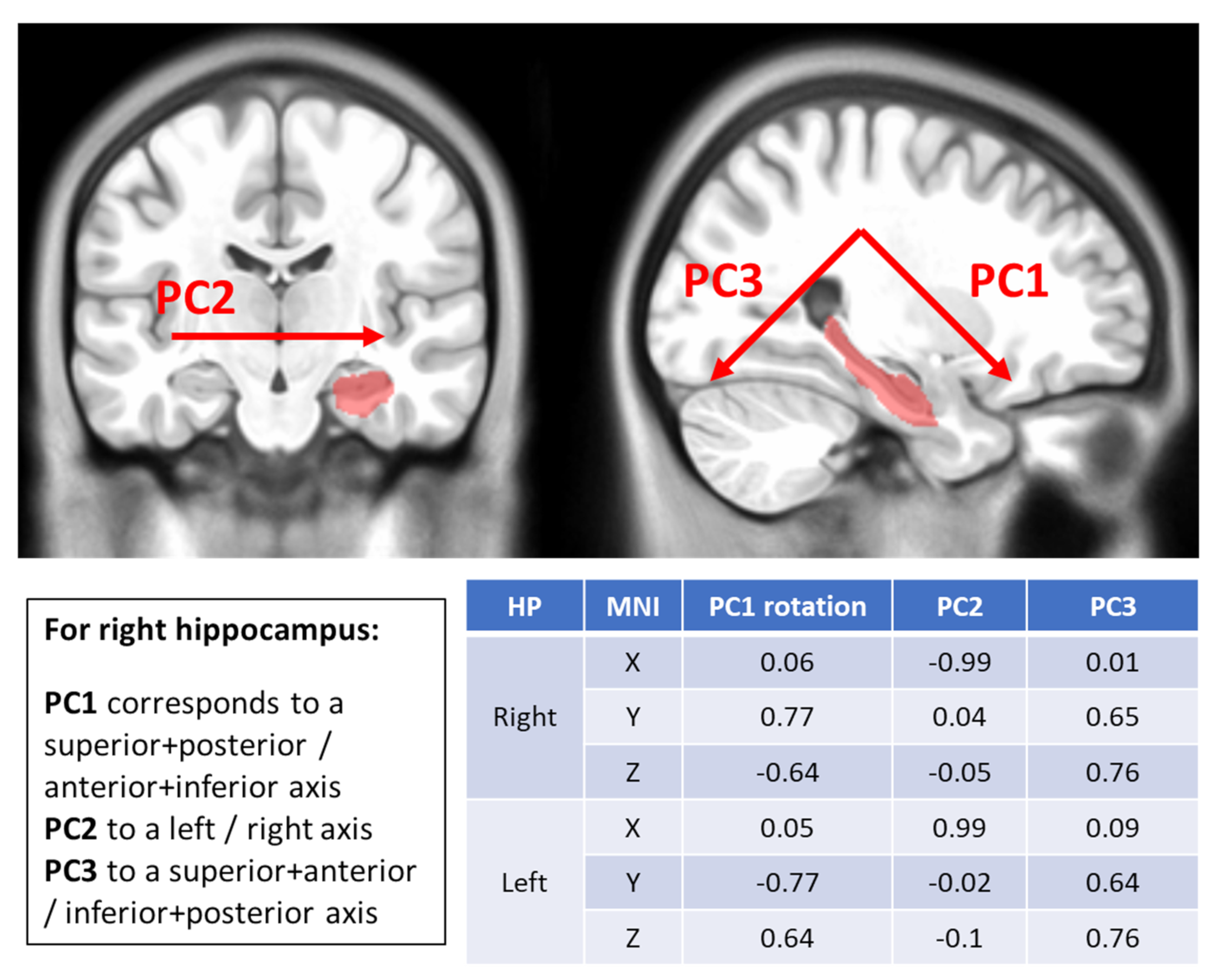

Supplement: Supplementary file 2 — Figure S1 [file 41398_2021_1310_MOESM2_ESM.tif]

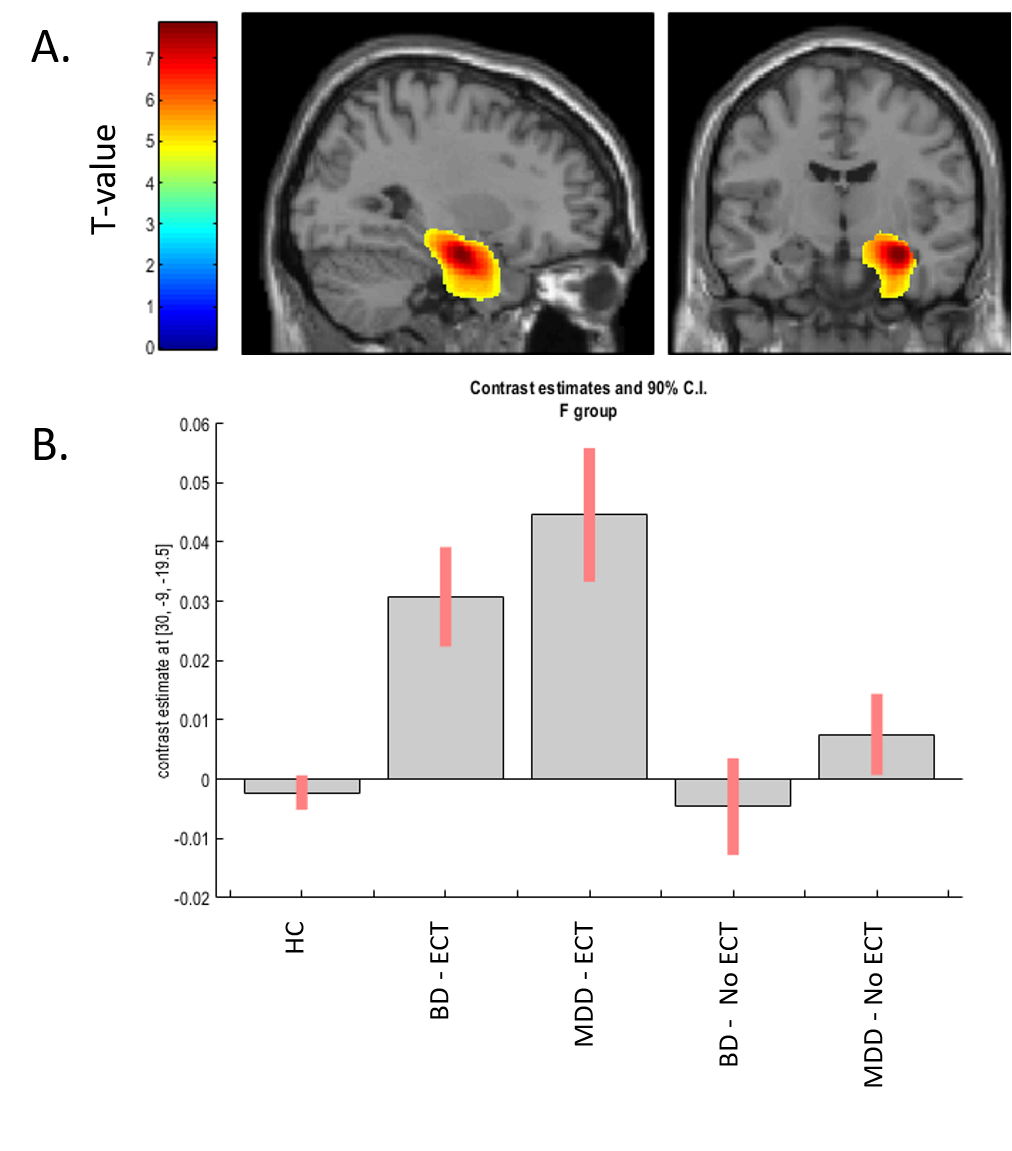

Supplement: Supplementary file 3 — Figure S2 [file 41398_2021_1310_MOESM3_ESM.tif]

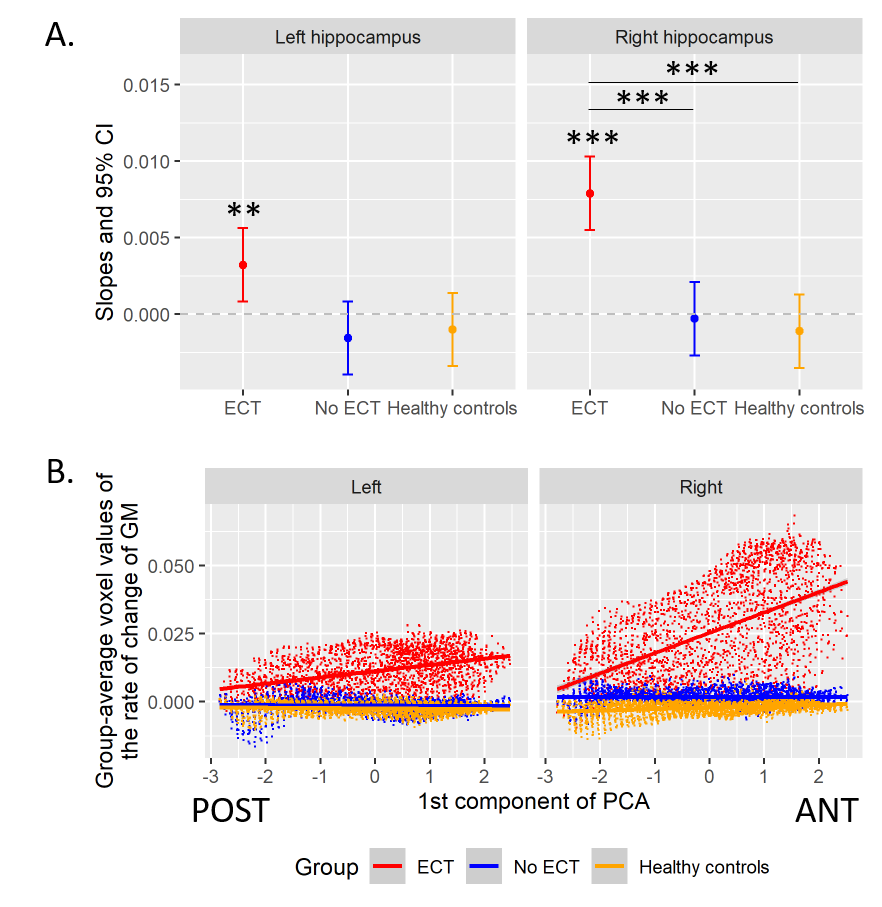

Supplement: Supplementary file 4 — Figure S3 [file 41398_2021_1310_MOESM4_ESM.tif]

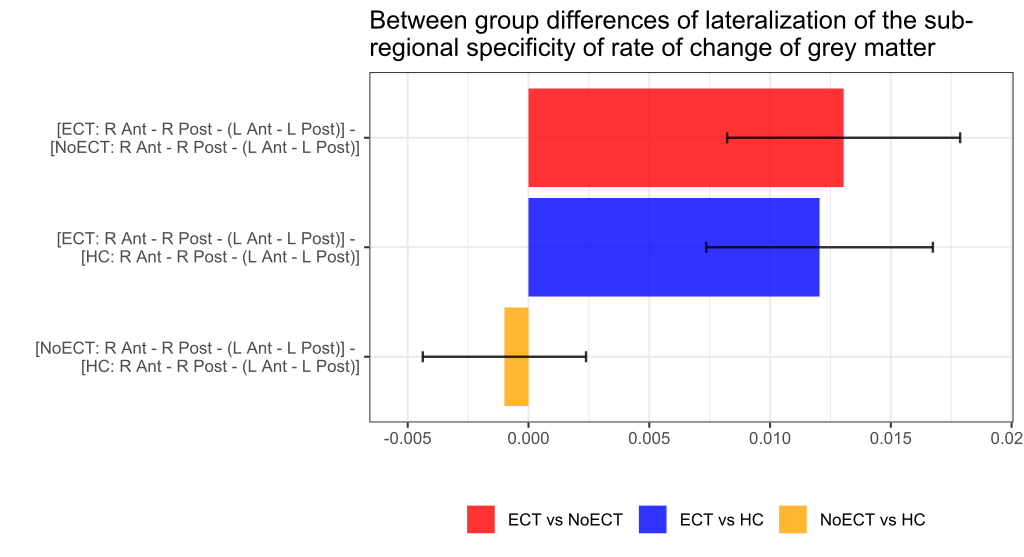

Supplement: Supplementary file 5 — Figure S4 [file 41398_2021_1310_MOESM5_ESM.tif]
